# Usage of a Multipurpose mHealth App Among Adults With Sickle Cell Disease: Randomized Controlled Trial

**DOI:** 10.2196/67906

**Published:** 2025-06-05

**Authors:** Robert M Cronin, Nives Quaye, Xin Liu, Kristina Landes, Lori E Crosby, Adetola A Kassim, Michael R DeBaun, Patrick M Schnell

**Affiliations:** 1 Department of Internal Medicine, The Ohio State University Wexner Medical Center, 410 W 10th Ave, Columbus, OH, 43210, United States, 1 6146889220; 2 Division of Biostatistics, The Ohio State University, Columbus, OH, United States; 3 Department of Pediatrics, Cincinnati Children's Hospital Medical Center, Cincinnati, OH, United States; 4 College of Medicine, University of Cincinnati, Cincinnati, OH, United States; 5 Department of Internal Medicine, Vanderbilt University, Nashville, TN, United States; 6 Department of Pediatrics, Vanderbilt University Medical Center, Nashville, TN, United States

**Keywords:** sickle cell disease, mHealth, patient engagement, hospitalizations, consumer health informatics, guidelines

## Abstract

**Background:**

While mobile health (mHealth) apps have been made for various diseases, including sickle cell disease (SCD), most focus on a single purpose. SCD is a chronic disease that requires knowledge of the disease, self-management, and adherence to treatment plans. While mHealth apps have been made with single features for SCD, there is limited understanding of using an mHealth app with a more comprehensive set of features that could engage adults with SCD, depending on what features they prefer and need to engage and empower them in living with their disease.

**Objective:**

We evaluated the usage of an mHealth app with various features, including pain tracking, quizzes for patient-facing guidelines, pain and asthma action plans, and goal setting.

**Methods:**

Adults with SCD were enrolled at 2 sickle cell centers between 2018 and 2022 as part of a 6-month feasibility randomized controlled trial with participants completing surveys at baseline and 6 months. Participants were randomized into receiving either an mHealth app and booklet with patient-facing guidelines or a booklet with the guidelines alone. The mHealth app comprised web pages with patient-facing guideline material and a Research Electronic Data Capture (REDCap) project. The REDCap project included a personal profile, a pain tracker, goal setting, quizzes about the guidelines, and pain or asthma action plans. The REDCap project also included the ability to send daily text messages at a time they chose, which contained a message they could create and a link to their profile. Outcomes included SCD-specific knowledge and acute health care utilization (emergency room visits and hospitalizations). We evaluated the usage of these different features and relationships with baseline variables, each other, and study outcomes.

**Results:**

Approximately 75% (50/67) of the enrolled and randomized participants completed all the study components, and 100% (26/26) of the participants who were randomized to the mHealth app arm and completed the study used the mHealth app. Further, 15/30 (50%) participants used multiple features. Baseline sickle cell knowledge and female gender were associated with more usage of pain diary (*P*=.04) and mission (*P*=.046) features, respectively. While not significant, mission completion was associated with lower hospitalizations (*P*=.06).

**Conclusions:**

Adults with SCD engaged differently with an mHealth app with multiple features. As this study was not focused on one part of our app, engagement with features in this app was entirely patient-driven, which may demonstrate the expected real-world use of an mHealth app in this population. A multipurpose app can help engage participants in self-management strategies through different features and potentially improve outcomes.

## Introduction

Sickle cell disease (SCD) is an inherited hemoglobin disorder that affects approximately 100,000 Americans, most of whom face considerable health care and health care technology disparities [1-4]. Over the last 40 years, SCD has changed from a life-threatening disease of early childhood to a chronic disease with multiple organ system involvement [5]. Individuals with SCD can develop issues with pain (acute and chronic), lung (asthma), heart, kidney, and mental health (depression). These complications lead to multifaceted treatment plans and the significant need for self-management. First, individuals with SCD can have acute vaso-occlusive pain episodes as well as chronic pain. These individuals can manage chronic and acute pain episodes using action plans like asthma. Second, disease-modifying medications need to be taken every day to prevent long-term sequelae and decrease pain. Finally, as with other chronic diseases, evidence-based guidelines advise health care providers about managing SCD and its complications [6]. We made them patient-facing to help individuals with SCD learn more about their disease and decide whether to act per the guidelines.

Many mobile health (mHealth) apps have been developed for SCD, but most focus on one aspect of the disease, primarily pain diaries [7-9]. No apps for SCD exist that combine components to be a multipurpose app that engages participants in multiple facets of their disease and their pain [10-12]. There has been no published evaluation of an mHealth app that encompasses various components of the disease process and allows individuals to tailor their needs to the mHealth app’s functionality.

**Figure 1.**
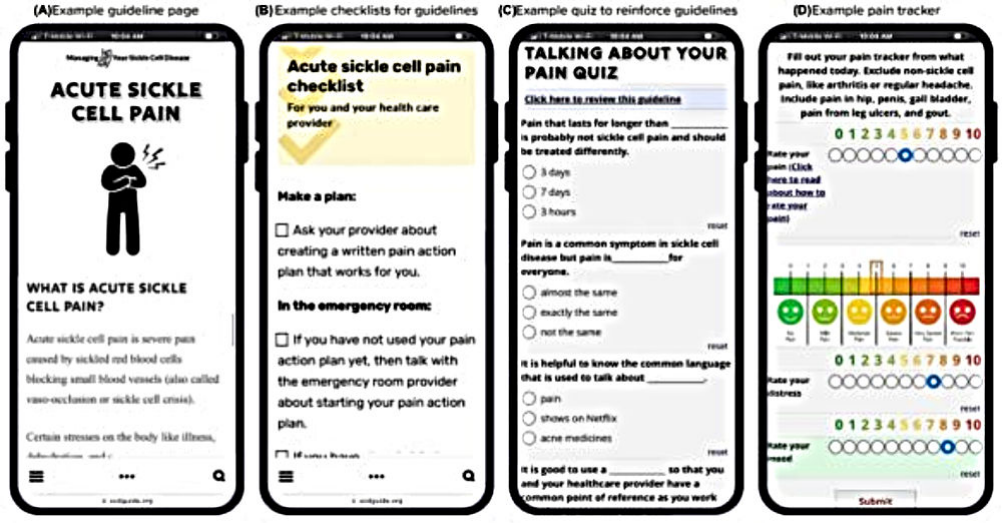
Screenshots from selected features of the mobile health app for adults with sickle cell disease used in this randomized controlled trial showing the (**A**) guideline, (**B**) checklist, (**C**) quiz, and (**D**) pain tracker.

We used the health belief model as our theory for health behavior decision-making to inform the design of our existing mHealth app for this study [13]. Our previous work described evidence-based recommendations that we included in our mHealth app, such as (1) guideline information that is easily accessible, understandable, and actionable; (2) alerts and cues, such as reminders, to promote awareness and adherence to health behaviors; (3) shared decision making including pain diaries, checklists of guidelines, and action plans to discuss with patient care teams; (4) decision support to help them understand the choices available to them in the guidelines, such as pain and asthma action plans, and (5) integration with personal preferences, for example, health goal setting (Figures1  2).

We conducted a feasibility randomized controlled trial to determine the acceptability of our mHealth app [14]. A multipurpose mHealth app can allow patients to monitor and track their pain, but additional features can increase app usage and engagement in their disease. As part of that trial, in this manuscript, we aimed to describe participants’ usage patterns and test the hypotheses that different usage would be associated with trial outcomes of knowledge of SCD-specific guidelines and hospitalizations and acute health care utilization.

**Figure 2.**
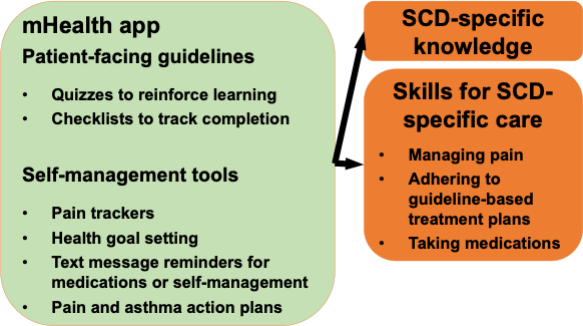
The mHealth app for adults with SCD used in this randomized controlled trial targets knowledge and skills for SCD through its patient-facing guidelines and self-management features. mHealth: mobile health; SCD: sickle cell disease.

## Methods

### Study Design

We conducted a parallel randomized controlled trial to assess feasibility as previously described [14] at 2 medical centers—Vanderbilt University Medical Center and the Ohio State University—from 2018 to 2022 (Checklist 1). Inclusion criteria encompassed patients within the SCD clinic with the following genotypes (Hemoglobin SS, Hemoglobin SC, and Hemoglobin Sβ-thal): proficient in spoken and written English, possessing a smartphone or computer, and being 18 to 70 years old. The trial ended due to reaching recruitment goals.

Participants were divided into two arms: (1) the mHealth app arm, where individuals received the mHealth app along with a booklet containing patient-facing guidelines, and (2) the booklet-only arm, where participants received solely a booklet with patient-facing guidelines. Participants were randomized in 1:1 ratio between the 2 arms without any restrictions. Randomization allocation sequence was determined by using a computer-generated random allocation sequence and implemented using the Research Electronic Data Capture (REDCap) randomization module.

The study involved an initial baseline and follow-up visits at 6 months. During the initial visit, after consenting, participants were randomized, and baseline surveys were administered by key study personnel. Participants in the mHealth app arm were introduced to the mHealth app, which took approximately 10‐15 minutes. At the 6-month follow-up, surveys were repeated, and all survey instruments were available electronically through REDCap, administered via iPads.

### Ethical Considerations

Institutional review boards at both centers—Vanderbilt University Medical Center (171036) and the Ohio State University (2020H0468)—approved the trial. Recruitment occurred at SCD clinics, and written informed consent was obtained before randomization. Compensation included a US $25 gift card for the initial visit and US $50 for the 6-month follow-up. Data were anonymized for analysis to help preserve privacy and confidentiality (MultimediaAppendices 1^4^).

### The mHealth App

The mHealth app comprised 2 main components: a web application featuring patient-facing guideline content and a REDCap project. The web application mirrored the content found in the previously mentioned booklets. It included sections providing guideline-based information on treatments (such as medications, transfusions, and transplants), complications (including acute sickle cell pain, infections, acute chest syndrome, pulmonary hypertension, leg ulcers, strokes, kidney disease, and depression), and wellness topics (women’s health, prevention and screening, pain triggers avoidance, stress management, dental care, diet and exercise, as well as tobacco, alcohol, and drug use).

The REDCap project featured a profile and interactive functionalities. Initially, participants could answer questions to generate their profiles, allowing them to access links for reviewing guidelines, editing their profiles, tracking pain, setting goals or missions, taking chapter quizzes related to the guidelines, and creating either a pain or an asthma action plan. The REDCap project also facilitated sending daily text messages at a time of the participant’s choosing, containing a customizable message and a link to their profile.

Missions were user-defined missions, goals, or tasks to complete during the trial. Specifically, participants could set a 6-month mission and as many weekly missions as they would like. Some examples of these missions included: “To better manage my pain without pain meds,” “Drink more water,” “No hospital visits,” “To Take all my medication on time each day,” “Recover from the hospital,” “To get a 9 to 5 job,” “Lose weight,” and “Establish and commit to a night routine to relax.”

Participants could document their daily pain experiences within the pain tracker and the strategies used to manage pain, including recording their mood. The goals feature enabled participants to monitor their weekly or 6-month goals. Chapter quizzes were available to reinforce the website’s content and the guidelines. The pain action plan guided individuals through a stepwise approach to managing acute pain at home. In contrast, the asthma action plan was designed for those seeking to develop a strategy for managing their asthma in collaboration with their health care provider. This mHealth app aimed to reinforce key guideline points and promote patient engagement through quizzes and text message reminders.

### Measurements of the mHealth App Usage and Outcomes

Primary and secondary outcomes were previously described [14]; in this manuscript, we evaluated mHealth app feature usage and associations with our outcomes. Pain diaries, goal-setting diaries, and quizzes were REDCap surveys, so we measured app feature usage based on the completion of these REDCap surveys. SCD knowledge outcome was measured with a 40-item questionnaire survey covering the guidelines during baseline and follow-up visits. Demographics were self-reported through surveys. Hospitalizations and acute health care utilization (hospitalization, emergency room visits, and day-hospital visits) outcomes were determined by chart review in the first 6 months following baseline and within 6 months prebaseline.

### Statistical Analysis

The sample for analysis of app usage was those who were randomized to the mHealth app arm, completed all baseline surveys, and either completed the study up to but not necessarily including the final endpoint survey or withdrew for a reason possibly related to the intervention (ie, not transplant or death). We used descriptive statistics, including median and IQR for continuous data and percentages for categorical data. Linear and generalized linear models evaluated baseline variables and usage differences between groups. Associations between usage of app components (diaries, quizzes, missions, and pain action plan) were evaluated using Wilcoxon test and Spearman correlations, as appropriate.

## Results

### Demographics and Overall App Usage

**Figure 3.**
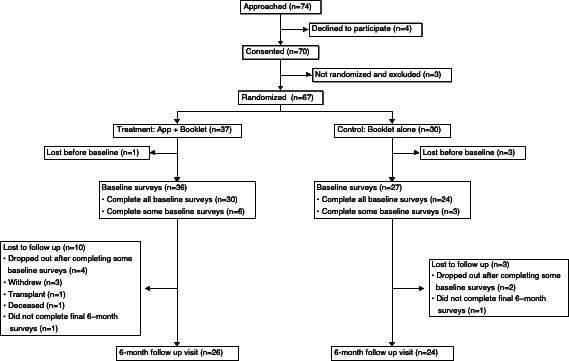
CONSORT (Consolidated Standards of Reporting Trails) flow diagram of the mobile health app for adults with sickle cell disease for the 6-month randomized controlled trial period.

A total of 67 participants were randomly allocated; 37 were enrolled in the mHealth app arm and 30 in the booklet alone arm (Figure 3). In total, 30 users who completed baseline surveys and were introduced to the mHealth app were eligible for app usage analysis: 26 completed the entire study, 3 withdrew after completing all baseline surveys but before the final follow-up survey, and 1 did not complete the final follow-up survey. There were no harms or unintended effects. Table 1 shows the baseline characteristics of the participants. All eligible users created a profile and filled out at least one pain diary. While there were no requirements to use the mHealth app, 90% (27/30) participants used at least one additional feature. Features in order of highest usage were missions (26/30, 87% of eligible participants), additional pain diaries (13/30, 43%), quizzes (8/30, 27%), and pain action plans (6/30, 20%). Further, 50% (15/30) participants used multiple features, not including the first pain diary (Table 2).

**Table 1.** Demographics of the 30 users who completed baseline surveys and were introduced to the mHealth app of this randomized controlled trial in adults with sickle cell disease.

Characteristics	Overall (n=30)
Age (years)	
Mean (SD)	34.5 (10.3)
Median (minimum-maximum)	32.0 [22.0-66.0]
Missing, n (%)	1 (3.3)
Racial identity, n (%)	
Black or African American	29 (96.7)
Missing	1 (3.3)
Ethnicity, n (%)	
Not Hispanic or Latino	25 (83.3)
Unknown or not reported	3 (10.0)
Hispanic or Latino	1 (3.3)
Missing	1 (3.3)
Gender identity, n (%)	
Man	16 (53.3)
Woman	12 (40.0)
Nonbinary	0 (0)
Prefer not to answer	1 (3.3)
Missing	1 (3.3)
Sexual orientation, n (%)	
Lesbian	1 (3.3)
Straight	24 (80.0)
Bisexual	2 (6.7)
Gay	1 (3.3)
Prefer not to answer	1 (3.3)
Missing	1 (3.3)
SCD^a^ type, n (%)	
Hemoglobin SS/Sbeta^0^ thalassemia	19 (63.3)
Hemoglobin SC/Sbeta^+^ thalassemia	10 (33.3)
Do not know	0 (0)
Missing	1 (3.3)
History of asthma, n (%)	
Yes	9 (30.0)
No	20 (66.7)
Missing	1 (3.3)
SCD knowledge score	
Mean (SD)	31.4 (4.47)
Median (minimum-maximum)	32.0 (18.0-37.0)
Missing, n (%)	2 (6.7)

a SCD: sickle cell disease.

**Table 2.** Feature utilization cross-tabulation among participants (adults with sickle cell disease) in this randomized controlled trial who were randomized to the app arm, completed baseline surveys, and were introduced to the mobile health app.

Feature	Total, n (%)	Nothing else, n (%)	Pain action plan, n (%)	Diaries, n (%)	Quizzes, n (%)	Missions, n (%)
Pain action plan	6 (20)	0 (0)	—^a^	5 (17)	6 (20)	6 (20)
Diaries	13 (43)	1 (3)	5 (17)	—	5 (17)	12 (40)
Quizzes	8 (27)	0 (0)	6 (20)	5 (17)	—	8 (27)
Missions	26 (87)	11 (37)	6 (20)	12 (40)	8 (27)	—

a Not applicable.

### App Function Usage and Association With Baseline Variables and Other App Purposes

#### Participants Had Different Patterns of Usage of Pain Diaries and Other Functions

**Figure 4.**
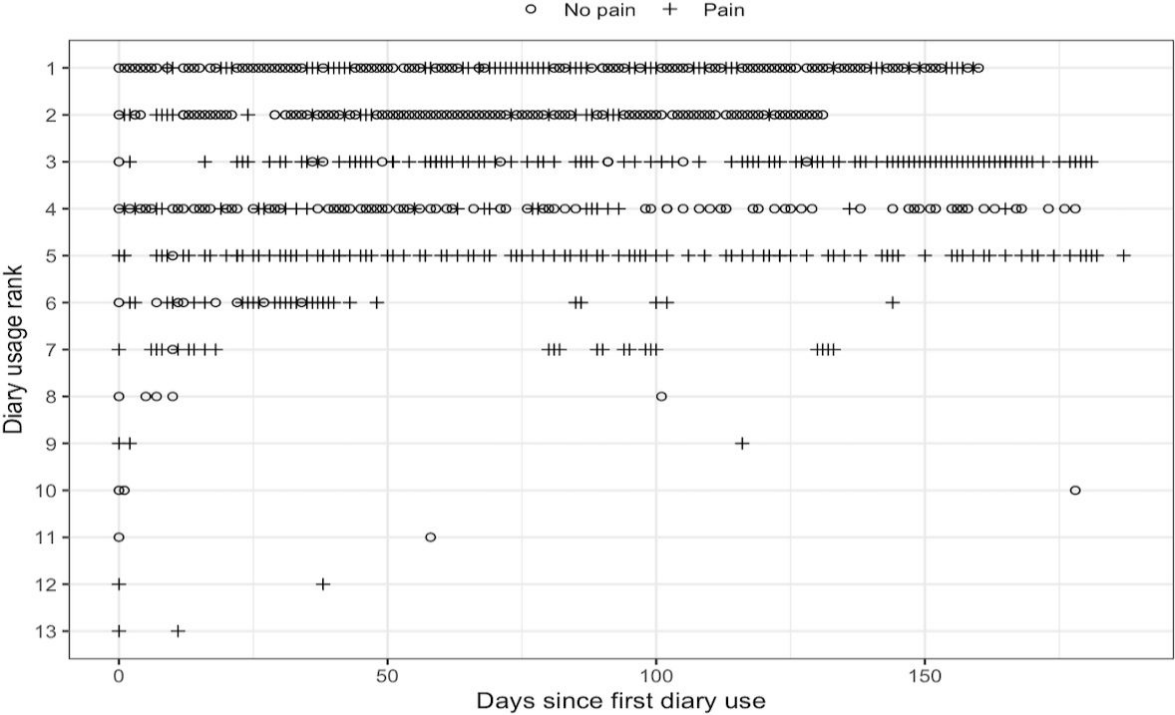
Pain diary usage of the mobile health app for adults with sickle cell disease for the 6-month randomized controlled trial period who completed more than one pain diary.

**Figure 5.**
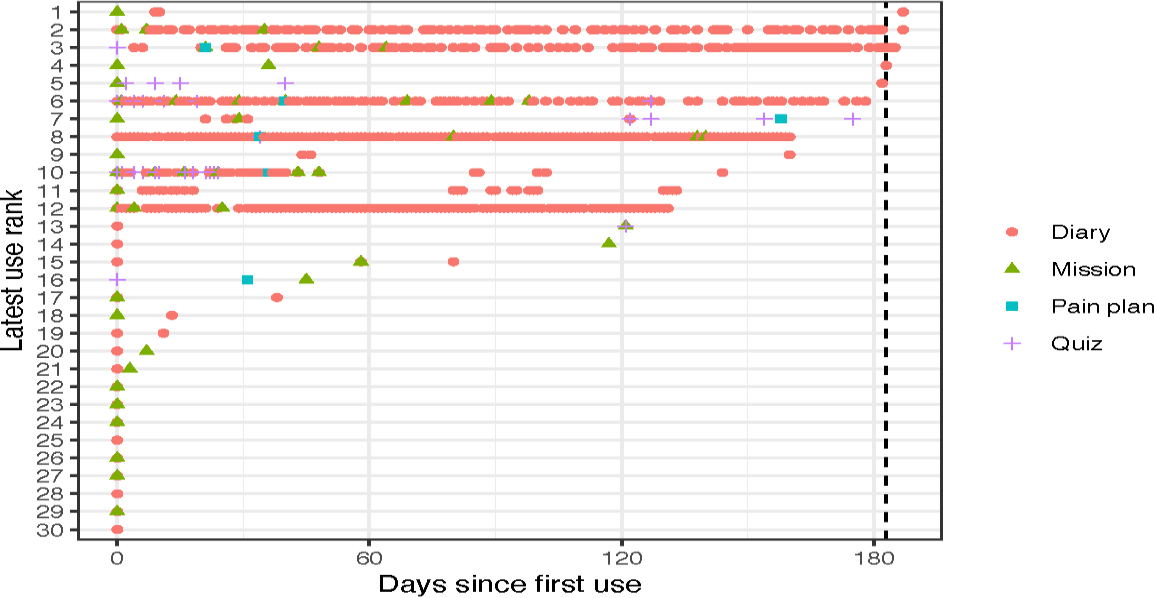
Different app feature usage of the mobile health (mHealth) app for adults with sickle cell disease who were randomized to the app arm, completed baseline surveys, and were introduced to the mHealth app for the 6-month randomized controlled trial period.

While a majority did not use the pain diary more than once (n=17), those who used the pain diary more than once had different patterns of use (Figure 4). Some used it almost daily (Figure 4, usage ranks 1‐5), measuring their chronic and sometimes acute pain. Others seem to only measure it from time to time (usage ranks 6‐7), possibly indicating they had no pain some of the time and pain other times, which could indicate days of chronic pain or acute pain exacerbations. Others used it more sporadically (usage ranks 8‐13), with one using it for about a week and then stopping (usage rank 8). In total, 21 of the 30 participants randomized to the app arm (70%) completed at least one more function after enrollment (Figure 5). Fourteen completed functions at 90 days (the half-way point of the trial), nine within the last month of the trial, but only 3 users completed functions after the trial was over.

#### Baseline Knowledge Was Associated With More Pain Diary Completion and Female Gender Identity with More Mission Completion

While baseline age, gender identity, disease type, SCD knowledge, and patient activation were not statistically significantly associated with most functions of the mHealth app, baseline knowledge was significantly associated with diary completion (Table 3). The more knowledge participants had at baseline, the more pain diaries they completed in addition to their first (33% increase per SCD knowledge point, 95% CI 6%-81%; *P*=.04). In addition, female versus male gender identity was statistically significantly associated with completing more missions (mean 3.8, SD 5.9 vs 1.2, SD 0.8; relative rate 317%, 95% CI 114%-1084%; *P*=.05).

**Table 3.** Associations between baseline variables and app usage for adults with sickle cell disease (SCD) who were randomized to the app arm, completed baseline surveys, and were introduced to the mobile health app for the 6-month randomized controlled trial period. Measures are relative rate (rr) for binary-count and continuous-count relationships, odds ratio (OR) for binary-binary relationships, and Cohen *d *for binary-continuous relationships.

	Diaries, rr (*P* value)	Pain plan, Cohen *d* or OR (*P* value)	Quizzes, rr (*P* value)	Missions, rr (*P* value)
Age	1.00 (1.00)	1.05^a^ (.15)	1.07 (.07)	1.00 (.90)
Gender (female vs male)	2.72 (.22)	3.34^b^ (.35)	1.52 (.67)	3.23 (.05)
SCD type^c^	0.35 (.32)	0.32^b^ (.63)	0.06 (.22)	0.42 (.27)
SCDK^d^	1.33 (.04)	0.60^a^ (.10)	1.09 (.55)	1.07 (.19)

a Value represents Cohen *d.*

b Value represents OR.

c Hemoglobin SC; Hemoglobin Sbeta-thalassemia+ vs Hemoglobin SS; Hemoglobin Sbeta-thalassemia0.

d SCDK: sickle cell disease knowledge.

#### Pain Action Plans, Pain Diaries, Quizzes, and Missions Were All Associated with Each Other

Patients who completed pain action plans completed more quizzes (median 5, IQR 2.5-6.74 vs 0, IQR: 0-0; *P*<.001), missions (median 3, IQR 2.25-5.25 vs 1, IQR 1-1; *P*=.003), and diaries (median 70.5, IQR: 12.8-111 vs 1.0, IQR: 1-2; *P*=.005) compared to those who did not (Table 4). Patients completing more missions tended to complete more diaries (rho=0.69; *P*<.001) and more quizzes (rho=0.47; *P*=.008), and those completing more diaries tended to complete more quizzes (rho=0.37; *P*=.047). Although the rank correlation between diaries and quizzes was positive, the relationship is more informatively described by clusters: most patients (n=23) completed few (0‐24) diaries and few (0‐4) quizzes, some (n=5) completed the most (100‐160 of 180 possible) diaries but few (0‐7) quizzes, and the remainder (n=2) completed the most (20 and 21 of 21 possible) quizzes but few (36 and 1) diaries. One patient was a notable outlier and completed the most missions (21, second-highest completed 8) and the second-most diaries (121, highest completed 157 and third-highest completed 113), but no quizzes or pain action plan.

**Table 4.** Associations between app usage features for adults with sickle cell disease who were randomized to the app arm, completed baseline surveys, and were introduced to the mobile health app for the 6-month randomized controlled trial period. Measures are Spearman rank correlations (rr) for count-count relationships and difference in medians (dm) for binary-count relationships.

	Diaries, dm or rr (*P* value)	Pain plan, dm or rr (*P* value)	Quizzes, dm or rr (*P* value)	Mission, dm or rr (*P* value)
Diaries	—^a^	69.5^b^ (.005)	0.4^c^ (.047)	0.7^c^ (<.001)
Pain plan	69.5^b^ (.005)	—	5.0^b^ (<.001)	2.0^b^ (.003)
Quizzes	0.4^c^ (.047)	5.0^b^ (<.001)	—	0.5^c^ (.008)
Missions	0.7^c^ (<.001)	2.0^b^ (.003)	0.5^c^ (.008)	—

a Not applicable.

b Value represents dm.

c Value represent rr.

We also evaluated the association of usage of different features with the trial outcomes, knowledge of SCD-specific guidelines and hospitalizations, and acute health care utilization, but the usage was not significantly associated with any outcomes (Table 5). A notable but not statistically significant relationship was observed between missions completed and hospitalizations. Of the 3 participants who had more hospitalizations in the 6 months of the trial than the 6 months before baseline, 2 completed no missions, and one completed 1 mission. In contrast, of the 5 participants with fewer hospitalizations during the trial than before, 3 completed one, 1 completed three, and one completed 21 missions, the highest of any participant. The Spearman rank correlation between missions completed and change in hospitalizations was rho=−0.38 (more missions were associated with fewer hospitalizations than pretrial; *P*=.05). Similar but weaker associations were observed between missions completed and hospitalizations due to pain (rho=−3.7; *P*=.06) and acute care utilization (rho=−0.31; *P*=.13).

**Table 5.** Associations between app usage and outcome for adults with sickle cell disease who were randomized to the app arm, completed baseline surveys, and were introduced to the mHealth app for the 6-month randomized controlled trial period. Measures are Spearman rank correlation (rc) for count-count and count-continuous relationships and difference in medians (dm) for binary-count and binary-continuous relationships.

	SCDK^a^	Hosp^b^	Acute care^c^
Diaries, rc (*P* value)	−0.17 (.43)	−0.16 (.43)	0.03 (.88)
Pain plan, dm (*P* value)	−0.16 (.47)	−0.08 (.69)	0.07 (.75)
Quizzes, rc (*P* value)	0.01 (.95)	−0.03 (.89)	0.18 (.39)
Missions, rc (*P* value)	−0.27 (.20)	−0.38 (.06)	−0.31 (.13)

a SCDK: sickle cell disease knowledge.

b Hosp: hospitalizations.

c Acute health care utilization (emergency room visits or hospitalizations).

## Discussion

### Principal Findings

mHealth apps are a potentially scalable, efficient, and sustainable strategy to improve adult care in SCD. However, there is a knowledge gap regarding patterns of the usage of multipurpose apps in rare diseases, such as SCD. Our study is the first to demonstrate that adults with SCD have different usage patterns of a multipurpose mHealth app for self-management, and certain features may be related to improved patient outcomes. Almost all users used multiple features. Highly engaged participants were more likely to use either quizzes or diaries, but not both. We also saw that completing certain features, such as missions, was associated with lower hospitalizations, although not significant. Complex chronic diseases, such as SCD, could benefit from multipurpose apps that allow users to engage based on preferences and needs.

As this study was not focused on one part of our app, engagement with features in this mHealth app was entirely patient-driven and may demonstrate the expected real-world use of an mHealth app in this population. Users were most engaged with missions but least engaged with action plans. Missions were one of the most straightforward, least time-consuming features that users could fill out independently, whereas action plans require more time and typically would be done with the provider. Interestingly, less than half of the participants used the pain diaries, potentially because they were not the primary function of the mHealth app or trial. This finding contradicts other studies that demonstrated the high use of pain diaries in SCD [8,15,16]. Usage of pain diaries in the general SCD population could be lower when it is not the primary goal of an mHealth app as part of a specific research study. Further study is needed into why some users completed pain diaries, among other mHealth app features, while others did not. While there were associations between the use of one feature of the mHealth app and another, participants tended to engage with some but not all features. For example, highly engaged participants engaged with either quizzes or diaries, but not both. Pain action plans, pain diaries, quizzes, and missions were all associated with each other. Almost all participants who engaged with one feature besides the missions would typically engage with additional features.

User engagement with mHealth apps is a critical area of study, as it directly influences the effectiveness of these interventions in promoting treatment adherence and self-management among individuals with chronic health conditions. Recent reviews have described challenges in engagement with mHealth apps, including (1) inconsistent terminology and measurements, limiting our ability to compare results across studies, complicating the development of effective engagement strategies [17]; (2) usability issues, where complex interfaces and poor user experiences in mHealth apps can deter individuals with chronic diseases from consistent use [18]; (3) data privacy and security concerns that can deter users from engaging with mHealth apps [19]; and (4) a lack of personalization, where generic content does not cater to individual user needs can lead to reduced engagement [20]. While our mHealth app included user-centered design to overcome usability issues and tailor the intervention to this population’s specific needs, is within REDCap, which has enhanced data privacy and security, and has personalization features, including text message reminders and individualized plans, the app’s current version had limited functionality to measure engagement robustly. By addressing these barriers and implementing the suggested strategies, developers and healthcare providers can improve user engagement with mHealth applications, leading to better health outcomes. Future research on user engagement could include post-study user interviews or focus groups to obtain more detailed information about why users engaged with certain functionality and not others.

A notable but not statistically significant relationship was observed between missions completed and hospitalizations. Findings from other studies demonstrated improved outcomes associated with apps [21]. However, while many studies show improvements in outcomes like asthma or diabetes control, few have demonstrated improvements in outcomes like hospitalizations. Also, others have shown the opposite effect of increased hospitalization rates with mHealth app usage [22,23]. Also, in adults with SCD, no studies demonstrated improvement in hospitalizations or acute health care utilization using mHealth apps. However, the feasibility study of our app showed a nonstatistically significant improvement in outcomes. A larger trial with more participants is the next logical step that could demonstrate which of the mHealth app’s features might improve outcomes.

Baseline knowledge and female gender identity were associated with more feature usage, whereas other variables, such as age, did not show any significant differences. Others have demonstrated that demographics, such as age, are associated with different usage [24] of an mHealth app; however, only female gender identity was associated with one more feature use. Classifying different types of users may be necessary for understanding and improving long-term engagement.

Our study had a low attrition rate; however, sustained engagement varied. Other studies have demonstrated a wide range of attrition rates from 9% to 82% and an average of 43% [25]. Our attrition rate was lower, which could be because our mHealth app used strategies that have led to lower attrition in other studies, including multitouch interventions and tailored messaging [25]. However, another reason for the low attrition could have been because our mHealth app had multiple features that allowed users to engage in the most important features to them and their disease For sustained engagement with the app, we observed that 70% of the users completed more functions after enrollment, with a continued decline throughout the study period, with only a few participants completing functions after trial completion. However, these features were not the primary feature of the trial, but we could not assess all app usage due to the app’s limited analytics. Future research with better analytics and a longer follow-up period is needed in understanding sustained usage of this mHealth app.

Since this mHealth app was built primarily in REDCap, the mHealth app can be accessed and used by any of the REDCap Consortium 7178 active partners across 156 countries. Providing a framework for an mHealth app built within REDCap using base features and external modules would demonstrate scalability by allowing researchers in the REDCap Consortium to efficiently adapt the mHealth app to develop, test, and confirm ideal configurations and settings for user engagement and data collection, which impact data quality and quantity and overall significance and applicability of study results. The consortium can download this mHealth app REDCap project and tailor it to their needs and local/national guidelines. Also, the solution would be more sustainable as it would not require maintenance and updates when operating systems change, like native Apple and Android apps, as the institutions typically update their versions of REDCap. However, for those outside of the REDCap consortium, there would be limitations on adapting this app quickly for their purposes, which would limit scalability to that app for nonconsortium partners.

Several limitations occurred in our study. First, the sample size was small, so we could not observe significant associations between mHealth app feature usage and outcomes. A larger randomized controlled trial would allow for further evaluation of the effects of important app features on outcomes. Second, we could not evaluate the interaction between participants and the guidelines feature of the mHealth app. While we measured how to access guidelines and other app features, we could not measure guideline review. This limited functionality also precluded us from being able to measure app usage after participants completed the trial. Our next version of the mHealth app will include analytics to evaluate guideline use. Third, the app’s current version did not include the ability to assess usage in terms of time, so we could not evaluate how long it took to perform different functions. Future hybrid effectiveness-implementation studies with better analytical functionality, a larger population, longer follow-up, and an implementation framework could help our understanding of improving outcomes, understanding user engagement, including sustained use after the trial is completed, and implementing this mHealth app.. Finally, this study did not assess other determinants of app usage (eg, participation in a community-based organization) or other healthcare experiences (eg, discrimination).

### Conclusions

Results of our first-ever multipurpose mHealth app for adults with SCD demonstrated that (1) participants will use an mHealth app and use certain features of an mHealth app depending on their needs, even if these features were not the primary purpose of the mHealth app, and (2) that certain features may lead to improvement in outcomes. A multipurpose mHealth app can help engage participants in features they want to use and potentially increase and improve outcomes. This trial’s results have important implications for improving the health of a patient population facing significant healthcare and health information technology disparities.

## Supplementary material

10.2196/67906 Multimedia Appendix 1 App usage data.

10.2196/67906 Multimedia Appendix 2 Data dictionary.

10.2196/67906 Multimedia Appendix 3 Subject deidentified data.

10.2196/67906 Multimedia Appendix 4 Prepost sickle cell disease knowledge scores.

10.2196/67906 Checklist 1 CONSORT (Consolidated Standards of Reporting Trails) checklist.

## Abbreviations

mHealth: mobile health
REDCap: Research Electronic Data Capture
SCD: sickle cell disease
